# Lower expression of endogenous intestinal alkaline phosphatase may predict worse prognosis in patients with Crohn’s disease

**DOI:** 10.1186/s12876-018-0904-x

**Published:** 2018-12-17

**Authors:** Seon-Young Park, Ji-Young Kim, Su-Mi Lee, Jin Ook Chung, Ji-Ho Seo, SunMin Kim, Dong Hyun Kim, Chang-Hwan Park, Jae-Kyun Ju, Young-Eun Joo, Jae-Hyuk Lee, Hyun-Soo Kim, Sung-Kyu Choi, Jong-Sun Rew

**Affiliations:** 1 Division of Gastroenterology and Hepatology, Chonnam National University Medical School, Gwangju, South Korea; 2 Division of Endocrinology and Metabolism, Chonnam National University Medical School, Gwangju, South Korea; 3 Department of Surgery, Chonnam National University Medical School, Gwangju, South Korea; 4 Department of Pathology, Chonnam National University Medical School, Gwangju, South Korea; 5 Department of Internal Medicine, Chonnam National University Medical School, 42, Jaebongro, Dong-ku, Gwangju, 501-757 Korea

**Keywords:** Inflammation, Recurrence, Crohn’s disease

## Abstract

**Backgrounds:**

Intestinal alkaline phosphatase (IAP) plays important role in gut homeostasis. We aimed to evaluate the expression of endogenous IAP and to assess the clinical course according to the expression of endogenous IAP in patients with Crohn’s disease (CD).

**Methods:**

A total of 32 consecutive patients (14 males) with CD were included in the study. We measured the level of endogenous iAP in inflamed and noninflamed colonic mucosa. To verify the inflammation status, we measured the level of mRNA for IL-6, TNF-α, and TLR-4. We monitored the clinical courses of patients during follow-up after acquisition of biopsy specimens.

**Results:**

Median age of patients was 22.5 years (range, 15–49). Median CD activity index (CDAI, range) was 93.7 (22.8~ 154.9). There were colonic involvements in all patients and perianal involvement in 43.8% patients. The mRNA levels of IL-6 (*p* = 0.005) and TLR-4 (*p* = 0.013) in inflamed mucosa were significantly higher than those in non-inflamed mucosa. However, there was no difference of expression of TNF-α mRNA (*p* = 0.345). During a 14-month follow-up (range, 9 months–54 months), there were 19 patients with clinical recurrences. There were 9 patients (9/19, 47.4%) with IAP expression ratio (inflamed to non-inflamed) ≤ 1.0 in patients with clinical recurrence while there was one patient (1/13, 7.7%) with IAP ratio ≤ 1.0 in patients without clinical recurrence (*p* = 0.024).

**Conclusion:**

Lower expression of IAP in inflamed mucosa compared to non-inflamed mucosa may be associated with clinical recurrence in patients with CD.

## Background

Intestinal alkaline phosphatase (IAP) is expressed by brush border of intestinal epithelium [1]. It plays an important role in the interaction between the gut flora and the host by detoxification of bacterial endotoxin, regulation of intestinal microbiome, and regulation of intestinal lipid absorption [2]. Low expression of IAP could increase the risk of disease through changes in the microbiome, inflammation, and permeability in intestine. Previous studies showed that low expression of IAP was associated with chronic inflammation-related diseases such as inflammatory bowel disease (IBD), celiac disease, obesity and insulin resistance [3–5]. Moreover, in mouse model with chronic colitis, IAP knockout mouse were more vulnerable to variable stimuli, suggesting protective roles of endogenous IAP for chronic inflammation [6]. Until now, there is limited information for the role of IAP as a predictor for prognosis of patients with Crohn’s disease (CD). We hypothesized that low expression of IAP may be associated with poor prognosis in subjects with CD. Here, we aimed to evaluate the clinical recurrences according to expression of endogenous IAP in colonic mucosa from patients with CD.

## Methods

### Subjects and specimen collections

We obtained biopsy tissue samples from the colonic mucosa during colonoscopy following informed consent (approved by the Ethics Committee of the Chonnam National University Hospital) in the 32 patients with CD. Biopsies were taken from grossly inflamed mucosa (edge of ulcer or aphthoid lesions if present), and from grossly non-inflamed mucosa in the same segment of colon. Colonic biopsy specimens were immediately snap-frozen in liquid nitrogen for protein analysis. Crohn’s disease activity index (CDAI) and SES-CD were evaluated around the time of colonoscopy. We monitored the clinical courses of patients during follow-up after acquisition of biopsy specimens.

### Evaluation of IAP expression

We performed quantification of IAP from each specimen using ELISA. We got colonic mucosal tissues using biopsy forceps and then homogenized those using the bead beater (QIAGEN Hilden, Germany) after protein extraction using the RIPA lysis buffer containing phosphatase inhibitor and protease inhibitor (sigma R0278, Sigma-Aldrich Co. LLC, USA). Protein concentrations of tissue lysates were quantitated by BCA™ protein assay kit (ThermoFisher scientific, USA) with a standard (bovine serum albumin). Levels of IAP were measured by ELISA using a human IAP ELISA kit (Cloud-Clone Corp., Uscn Life Science Inc., Houston, USA) in which microtiter plate has been precoated with specific antibody to IAP. Absorbance was measured at 450 nm using a VersaMax™ ELISA reader (MOLECULAR DEVICES, Silicon Valley, USA). We performed all reactions in duplicates and calculated the average value of duplicate results. The minimum detectable dose of IAP was typically less than 0.63 ng/mL.

### RNA isolation and real-time polymerase chain reaction

To compare the degree of inflammation between the inflamed lesion and the noninflamed lesion, we determined the mRNA levels of pro-inflammatory genes such as IL-6, TNF-α and TLR-4 by real-time polymerase chain reaction (PCR) Taqman probe assay. Total RNA was isolated using 1 mL Trizol reagent (Takara Bio Inc. Japan) according to RNA extraction manufacturer’s protocol. We subsequently converted total RNA to cDNA using the Reverse Transcription System (Promega, Wisconsin, USA). And then we analyzed mRNA of inflammatory factors by the real time PCR. We used Thermo Scientific™ commodified Taqman probe and primer set for inflammatory cytokine mRNA level detection by real time PCR. Catalogue number of Cytokines probe and primer set were HS02758991-g1:GAPDH, HS00174131-m1:IL-6, HS01113624-g1:TNF, HS00152939-m1:TLR4(Thermo Fisher Scientific inc.). We detected the products using the ABI PRISM 7900HT Detection system (ThermoFisher scientific, USA). We calculated quantitative change of relative gene expression with 2^-ΔΔCT^ method. The house keeping gene is glyceraldehyde phosphate dehydrogenase (GAPDH).

### Outcomes

The primary outcome was clinical recurrence that was defined as change of prescription, bowel resection, fistulotomy, stricture plasty, stoma formation, CD-related hospitalization or flare during follow-up period. CD-related hospitalization was defined as cases resulting from complications including the following: CD-related surgery, hospitalization for nonsurgical CD-related events, such as CD-related flares; hospitalization related to complications/extraintestinal manifestation of CD and disease flare.

### Statistical analysis

We used Wilcoxon signed rank test (one sample nonparametric test) to compare the expression of inflammatory markers and the IAP between inflamed mucosa and noninflamed mucosa in patients with CD. Mann-Whitney U test was used to compare the level of endogenous IAP between patients with clinical recurrence and patients without clinical recurrence during follow-up. The Statistical Package for the Social Sciences (SPSS)/PC 20.0 (Chicago, IL, USA) was used to perform the calculations. Differences were considered significant at *p* < 0.05.

## Results

### Demographics and baseline clinical characteristics

A total of 32 consecutive patients (22 males) with CD were in clinical remission when they were included in the study. Median age (range) of patients was 22.5 years (15 years–49 years). Median CDAI (range) was 93.7 (22.8~ 154.9). All patients had undergone colonoscopy for surveillance of CD. There were colonic involvements in all patients and perianal involvement in 43.8% patients (Table 1).

**Table 1:** Demographics of 32 patients with Crohn’s disease

	*N* = 32	Patients without clinical recurrences (*n* = 13)	Patients with clinical recurrences (*n* = 19)	*p*-value
Male, *n* (%)	22 (68.8)	10 (76.9)	12 (63.2)	0.467
Age at inclusion, median (range), yrs.	22.5 (15–49)	23.0 (15~ 49)	22.0 (15~ 38)	0.260
Clinical symptoms, *n* (%)				
diarrhea	18 (56.3)	9 (69.2)	9 (47.4)	0.289
Abdominal pain	13 (40.6)	5 (38.5)	8 (42.1)	0.837
Bloody stool	8 (25.0)	2 (15.4)	6 (31.6)	0.299
Mucoid stool	2 (6.3)	0 (0.0)	2 (10.5)	0.502
fever	1 (3.1)	0 (0.0)	1 (5.3)	0.594
Weight loss	3 (9.4)	1 (7.7)	2 (10.5)	0.999
Disease, phenotype, *n* (%), Montreal classification				
L1 (isolated ileal type)	0 (0)	0 (0)	0 (0)	0.473
L2 (isolated colonic type)	12 (37.5)	6 (46.2)	6 (31.6)	
L3 (ileocolic type)	20 (62.5)	7 (53.8)	13 (68.4)	
B1 (nonstricturing, nonpenetrating)	4 (12.5)	1 (7.7)	3 (15.8)	0.528
B2 (strcituring)	17 (53.1)	7 (53.8)	10 (52.6)	
B3 (penetrating)	11 (34.4)	5 (38.5)	6 (31.6)	
P (concomitant perianal disease)	14 (43.8)	4 (30.8)	10 (52.6)	0.221
Laboratory findings, median value (range)				
WBC, median value (range)	7100 (2700–13,400)	7100 (5000–12,600)	6900 (2700–13,400)	0.929
Hb	12.9 (9.0–16.0)	13.6 (10.3–16.0)	12.9 (9.0–16.0)	0.421
Serum albumin	4.1 (3.0–5.0)	4.1 (3.4–5.0)	4.1 (3.0–5.0)	0.453
C-reactive protein	1.74 (0.68–3.71)	2.13 (0.05–7.09)	1.73 (0.03–7.93)	0.764
Medication, *n* (%)				
5-Aminosalicylic acid	28 (87.5)	11 (84.6)	17 (89.5)	0.542
Corticosteroid	0 (0.0)	0 (0.0)	0 (0.0)	0.999
Azathioprine	12 (37.5)	4 (30.8)	8 (42.1)	0.393
TNF-α antagonists	2 (6.3)	0 (0.0)	2 (10.5)	0.345
CDAI, median (range)	93.7 (22.8~ 154.9)	84.9 (22.8~ 154.9)	95.9 (46.1~ 147.1)	0.387
SES-CD, median (range)	14.0 (4~ 37.0)	13.0 (4.0~ 31.0)	14.0 (4.0~ 37.0)	0.486

*CDAI* Crohn’s Disease Activity Index, *SES-CD* Simplified endoscopic score of Crohn’s disease, *TNF- α* tumor necrosis factor alpha

### Expression of pro-inflammatory genes between the non-inflamed mucosa and inflamed mucosa in patients with Crohn’s disease

**Fig. 1:**
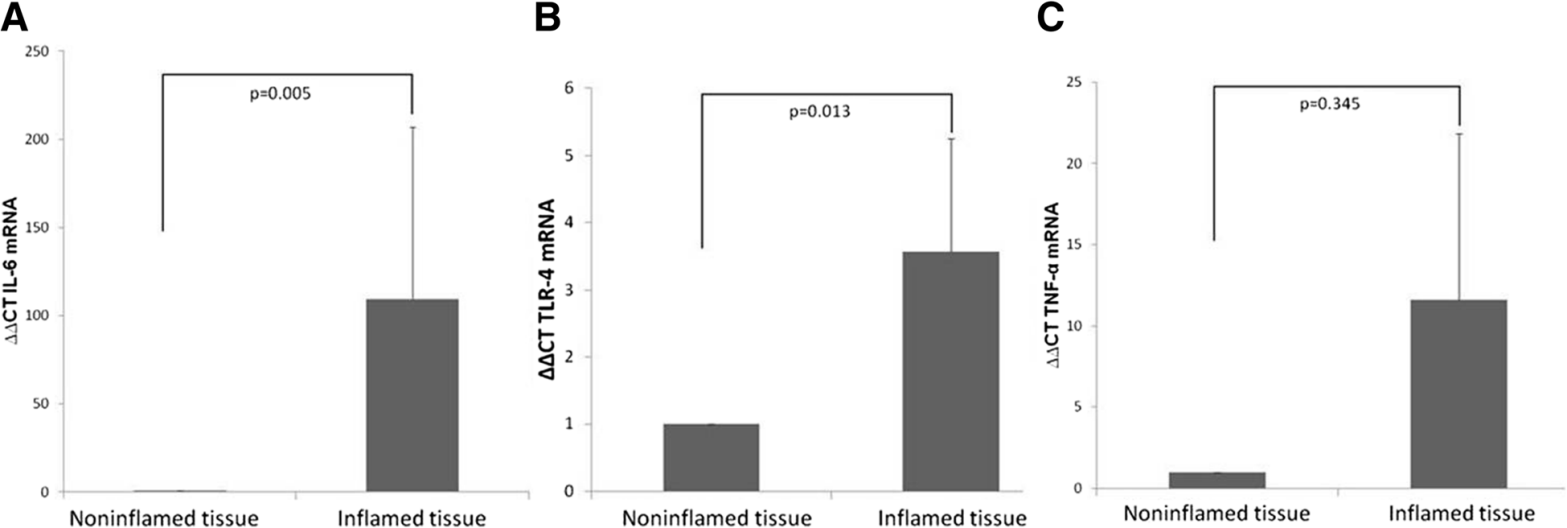
Expression of pro-inflammatory genes between the inflamed mucosa and non-inflamed mucosa in patients with Crohn’s diseases. Expression of mRNA levels of IL-6 (**a**) and TLR-4 (**b**) in inflamed mucosal were higher than those in non-inflamed mucosa. There was no significant difference in TNF-α (**c**)

To verify the degree of inflammation between the inflamed and non-inflamed mucosa, we evaluated the mRNA levels of inflammatory genes. The mRNA levels of IL-6 (*p* = 0.005) and TLR-4 (*p* = 0.013) in inflamed mucosa were significantly higher than those in non-inflamed mucosa. However, there was no difference of expression of TNF-α mRNA (*p* = 0.345, Fig. 1).

### Clinical course in patients with Crohn’s disease

During a 14-month follow-up (range, 9 months–54 months), there were 19 patients with clinical recurrences. There were 9 patients who need a change of prescription, 3 patients who had undergone bowel resection, 6 patients who had undergone fistulotomy, and 5 patients who were hospitalized for complications/extraintestinal manifestation of CD and disease flare.

Table 1 showed the differences of prognostics between patients with clinical recurrences and patients without clinical recurrences. There were no significant differences in clinical symptoms, disease phenotype, baseline laboratory findings, CDAI and SES-CD between 2 groups.

### Expression of endogenous IAP protein between inflamed mucosa and non-inflamed mucosa in patients with Crohn’s disease

**Fig. 2:**
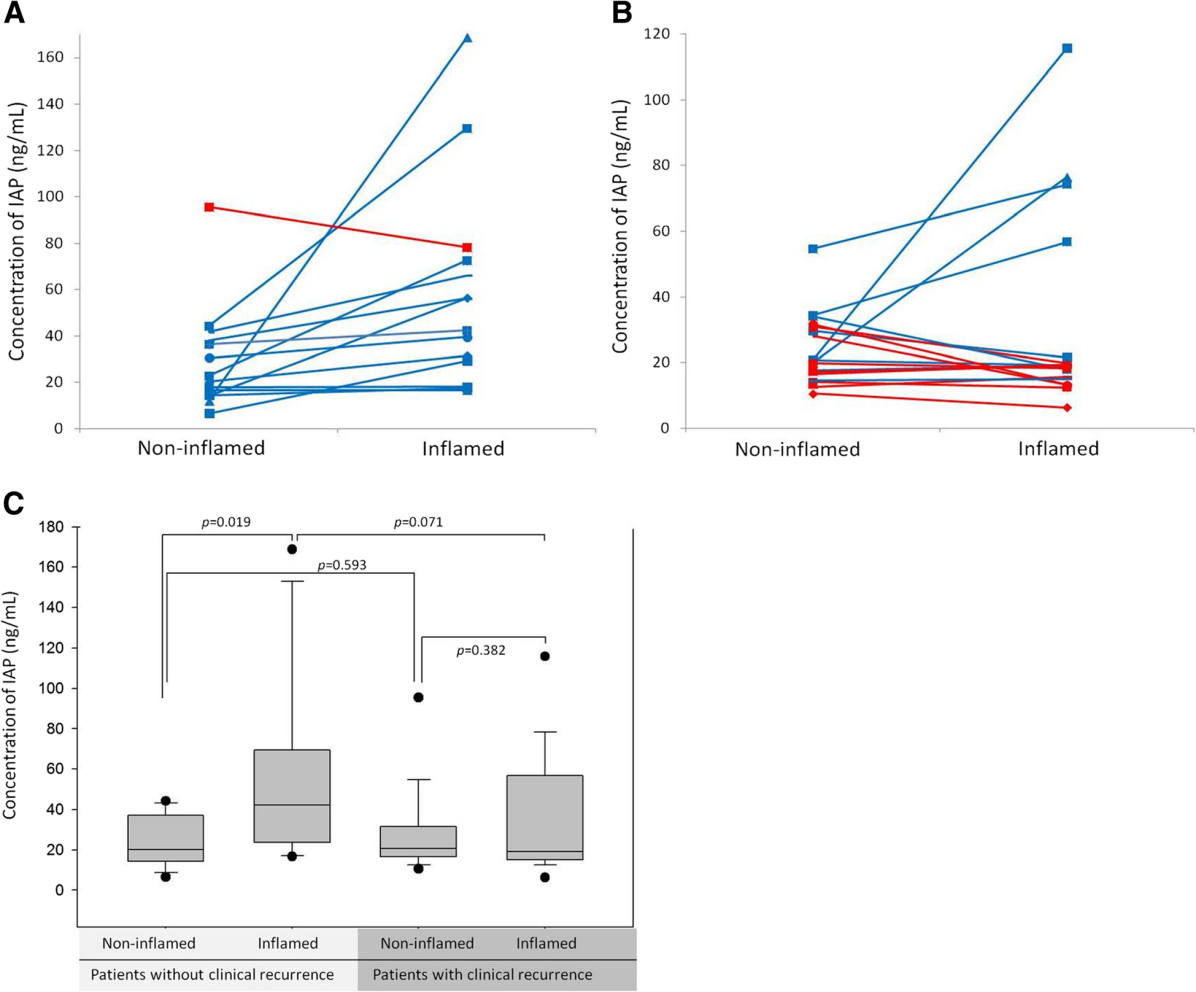
Concentration of IAP in colonic mucosa from patients without clinical recurrence and with clinical recurrence. **a** Only one patient without clinical recurrence had lower concentration of IAP from inflamed mucosa compared to non-inflamed mucosa. **b** Among 19 patients with clinical recurrence, 47.4% patients had lower concentration of IAP from inflamed mucosa compared to inflamed mucosa. **c** In patients without clinical recurrence, expression of IAP from inflamed mucosa was higher than that from non-inflamed mucosa (p = 0.019). However, in patients with clinical recurrence, there was no significant difference in IAP between noninflamed mucosa and inflamed mucosa

Mean (SD) concentrations of IAP in non-inflamed mucosa and inflamed mucosa were 26.3 (16.8) ng/mL and 43.0 (38.6) ng/mL, respectively. There were no significant differences in concentration of IAP of non-inflamed mucosa and inflamed mucosa between patients with clinical recurrences and patients without clinical recurrences (both *p* > 0.05). However, in patients without clinical recurrence, expression of IAP from inflamed mucosa was higher than that from non-inflamed mucosa (*p* = 0.019). In patients with clinical recurrence, there was no significant difference in IAP between noninflamed mucosa and inflamed mucosa (Fig. 2). There was no correlation between concentrations of IAP in non-inflamed mucosa and SES-CD (rho = − 0.08, *p* = 0.672). There was no correlation between concentrations of IAP in inflamed mucosa and SES-CD (rho = − 0.11, *p* = 0.540).

The median ratio of IAP concentration of inflamed mucosa to non-inflamed mucosa in patients was 1.2 (range, 0.4–13.9). There were 10 patients with IAP ratio ≤ 1.0 and 22 patients with IAP ratio > 1.0. There was a significant difference in clinical recurrences based on IAP ratio (≤1.0). There were 9 patients (9/19, 47.4%) with IAP ratio ≤ 1.0 in patients with clinical recurrence while there was one patient (1/13, 7.7%) with IAP ratio ≤ 1.0 in patients without clinical recurrence (*p* = 0.024, Fig. 2).

## Discussion

In this study, we showed that 31.3% patients had relatively lower IAP expression in inflamed colonic mucosa compared to noninflamed colonic mucosa. Among those, 90% experienced clinical recurrences during follow-up.

There were inconsistent results for IAP activity in inflammatory bowel condition. IAP activity in inflamed tissue was marked upregulated in multiple models of colitis [7]. However, decreased IAP expression has been known to be involved in many chronic inflammatory diseases such as IBD, celiac disease, antibiotic associated diarrhea and necrotizing enterocolitis [8]. Furthermore, recent studies suggested a therapeutic role of human recombinant IAP in the management of chronic inflammatory bowel diseases [4, 9]. Therefore, we hypothesized that relative expression of IAP in inflamed mucosa compared to noninflamed mucosa may be associated with prognosis in patients with CD. In our study, 22 patients had higher expression of endogenous IAP in inflamed colonic mucosa compared to noninflamed colonic mucosa (ratio of endogenous IAP > 1.0). On the contrary, 10 patients had lower expression of endogenous IAP in inflamed colonic mucosa compared to noninflamed colonic mucosa (ratio of endogenous IAP ≤ 1.0), which was similar with previous other studies [4, 10]. We demonstrated that aggravation of underlying disease activity, which needed to change managements, had more frequently occurred in patients with low ratio (≤1.0) of endogenous IAP expression than in patients with high ratio (> 1.0) of endogenous IAP. These finding suggest the role of endogenous IAP expression as prognostic factors for patients with CD.

Clinical assessment is a critical for patients care, though several biomarkers and radiologic and endoscopic scoring system have a role to assess disease activity, determine prognosis, and predict response to therapeutic strategies. There are available use biomarkers including fecal calprotectin, C-reactive protein, erythrocyte sedimentation rate, peripheral antinuclear cytoplasmic antibody (p-ANCA), anti-Saccharomyces antibody (ASCA), and serum albumin in real clinical practice. In our study, we showed that there were no significant differences in laboratory findings, endoscopic scoring system, and CDAI between patients with clinical recurrence and patients without clinical recurrence [11]. Furthermore, there was no correlation between concentrations of IAP and clinical recurrence, which may limit the potential utility of absolute concentration of IAP as a prognostic marker. However, a low ratio of endogenous IAP (< 1.0) was correlated with poor prognosis in our study. Therefore, relative expression of IAP rather than absolute value of IAP may be more useful for prediction of prognosis.

There were several reports showing the effectiveness of exogenous IAP in management of IBD [6]. Several studies reported therapeutic effects of exogenous oral IAP in experimental colitis model [2, 12–14]. In colitic models, exogenous administration of IAP improved the macroscopically and microscopically finding of colonic inflammation through inhibition of LPS-induced production of TNF-α and IL-6 and TLR-4 dependent pathway [4, 6, 15, 16]. Administration of calf IAP by oral route prevented weight loss in IAP-knockout mice and dextran disulfate sodium induced colitis model. In patients with moderate to severe ulcerative colitis, enteral administration of IAP improved short-term clinical course [4]. Therefore, endogenous IAP may affect mucosal protection in patients with CD. In our study, incidence of clinical recurrence was higher in patients with lower expression of endogenous IAP compared to patients with higher expression of endogenous IAP.

Limitations of our study were as follows; (1) most patients in our study had moderate disease activity during enrollment period. That is one of plausible reasons why the markers including concentration of IAP didn’t predict the outcomes. Therefore, it will be difficult to impact current care practices with current results. (2) we couldn’t get the information of IAP expression during clinical recurrence as ethical problem.

## Conclusions

Relatively lower expression of IAP in inflamed mucosa compared to noninflamed mucosa may be associated with poor prognosis. In the future, it is necessary to perform study with larger sample size and variable disease activities to evaluate the role of IAP as a prognostic factor. And, the effectiveness of tailored treatment of IAP based on the expression of endogenous IAP need to be studied.
